# Catchment-scale biogeography of riverine bacterioplankton

**DOI:** 10.1038/ismej.2014.166

**Published:** 2014-09-19

**Authors:** Daniel S Read, Hyun S Gweon, Michael J Bowes, Lindsay K Newbold, Dawn Field, Mark J Bailey, Robert I Griffiths

**Affiliations:** 1 Centre for Ecology & Hydrology, Wallingford, UK

## Abstract

Lotic ecosystems such as rivers and streams are unique in that they represent a continuum of both space and time during the transition from headwaters to the river mouth. As microbes have very different controls over their ecology, distribution and dispersion compared with macrobiota, we wished to explore biogeographical patterns within a river catchment and uncover the major drivers structuring bacterioplankton communities. Water samples collected across the River Thames Basin, UK, covering the transition from headwater tributaries to the lower reaches of the main river channel were characterised using 16S rRNA gene pyrosequencing. This approach revealed an ecological succession in the bacterial community composition along the river continuum, moving from a community dominated by Bacteroidetes in the headwaters to Actinobacteria-dominated downstream. Location of the sampling point in the river network (measured as the cumulative water channel distance upstream) was found to be the most predictive spatial feature; inferring that ecological processes pertaining to temporal community succession are of prime importance in driving the assemblages of riverine bacterioplankton communities. A decrease in bacterial activity rates and an increase in the abundance of low nucleic acid bacteria relative to high nucleic acid bacteria were found to correspond with these downstream changes in community structure, suggesting corresponding functional changes. Our findings show that bacterial communities across the Thames basin exhibit an ecological succession along the river continuum, and that this is primarily driven by water residence time rather than the physico-chemical status of the river.

## Introduction

Lotic environments such as rivers and streams are unique ecosystems in that they represent a continuum of both space and time during the transition from headwaters to the river mouth. This is accompanied by significant downstream hydrological and biogeochemical changes and a succession of biotic communities. In many rivers there is a change in the nature of inputs from both natural and anthropogenic sources during this transition. In addition, fluvial networks differ from most terrestrial ecosystems in that biological dispersal is limited, where landscape structure and physical flows determine the distance and direction of movement (Altermatt, 2013; Mari *et al.*, 2014). The downstream gradient in riverine communities has been described by the River Continuum Concept, that explains the response of the structure and function of river biota to the gradient of physical factors (physico-chemical and hydrological) within the catchment (Vannote *et al.*, 1980). The concept describes how riverine biotic assemblages exhibit longitudinal shifts in response to upstream processes and changing hydromorphology. The concept also postulates that the majority of organic inputs in the higher reaches of the catchment are derived from allochthonous sources such as leaf litter and from autochthonous production by phytoplankton in the lower reaches. Further developments to this concept have included an increased weighting of the role of flooding (Junk *et al.*, 1989), and autochthonous production from phytoplankton, benthic algae and plants (Thorp and Delong, 1994) in structuring aquatic ecosystems.

The relationship between the longitudinal dimensions of a river (that is, distance downstream) and biological diversity is not always clear and can depend upon the river system and the organisms being examined. Studies on fish have shown strong relationships of increasing biological diversity with decreasing distance to the river outlet (Muneepeerakul *et al.*, 2008) and this relationship has been linked to river discharge (McGarvey and Ward, 2008). Research on invertebrates has shown similar patterns, with positive relationships between species richness and catchment area (Altermatt *et al.*, 2013). However, there is evidence that these patterns may not be universal (Perry and Schaeffer, 1987), leading to more complex patch (Thorp *et al.*, 2006)- and network (Altermatt, 2013)-based models in an attempt to describe patterns of lotic biocomplexity.

For bacterial communities the relationship between diversity and geographic space has been well studied; with a particular focus on soil bacterial communities at a range of scales, from field studies (Sayer *et al.*, 2013) to country-scale surveys (Griffiths *et al.*, 2011; Ranjard *et al.*, 2013), but also including work on aquatic environments, from rock pools (Langenheder *et al.*, 2012) to lakes (Logue *et al.*, 2012). However, lotic environments such as rivers represent an understudied and unusual habitat owing to their constant turnover and therefore rules relevant to more static habitats such as soils or even lentic environments may not apply. In addition, microorganisms differ from many macroorganisms in that they are generally passive dispersers (Nemergut *et al.*, 2013), with the direction of dispersal determined by the movement of water. Rivers and streams also have a large number of potential inputs, each adding new microbial communities to the mix. For example, although at any point in a fluvial system the majority of the water, and therefore microbial community, is likely to have originated from upstream, additional inputs can arrive from groundwater (Sorensen *et al.*, 2013), soil and surface runoff (Crump *et al.*, 2007), atmospheric and precipitation inputs (Christner *et al.*, 2008; DeLeon-Rodriguez *et al.*, 2013) and anthropogenic point sources such as sewage outlets (Newton *et al.*, 2013). Moreover, many fluvial networks worldwide are highly modified by land use changes and the connection of man-made waterways such as canals and reservoirs, causing significant changes in water chemistry, flow velocities and water residence times (Whitehead *et al.*, 2013).

Gaining a better understanding of the spatial and temporal patterns of freshwater microbial diversity, their major drivers and their resistance and resilience to environmental change is of critical importance. Microbes are involved with a large array of ecological process in fluvial systems, from recycling, releasing, storing and transforming nutrients (Tatariw *et al.*, 2013), biogeochemical processes linked to climate change (Butman and Raymond, 2011; Raymond *et al.*, 2013), persistence and transport of pathogens (Marti *et al.*, 2013) to acting as reservoirs of antibiotic resistance (Marti *et al.*, 2014).

This study addresses the fundamental question of how bacterioplankton community composition varies across a major river basin (the River Thames in England) subject to wide gradients in physical conditions and anthropogenic pressures. By revealing successional patterns in the composition and function of bacteria across a river basin, we have identified a significant physical control over the structure of free-living riverine bacteria, setting a framework for future research on this topic.

## Materials and methods

### Sampling and contextual environmental variables

**Figure 1:**
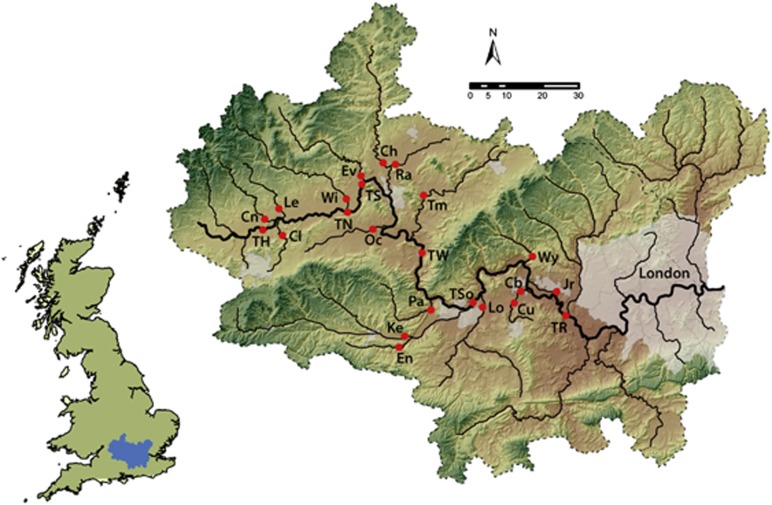
A map of the River Thames basin showing the sampling sites used in this study. Lines indicate the main river channel and do not include higher order tributaries for clarity.

The study site was the River Thames basin in southern England, UK. The River Thames is the largest river completely in England, with a total length of 354 km to its tidal limit and a catchment area of 9948 km^2^ (Marsh and Hannaford, 2008) (Figure 1). Although the basin has a high population density (960 people per km^2^), much of the upstream catchment is relatively rural, comprising mainly arable crops and grassland (Bowes *et al.*, 2014). Samples were taken from 23 monitoring sites, as part of the Centre for Ecology and Hydrology's Thames Initiative research platform, during low flow conditions on 13 September 2011. The sampling sites were located on all major tributaries joining the River Thames between Hannington Wick (site TH) in the upper Thames basin and Runnymede (site TR) on the outskirts of London, and also six sites along the main channel of the River Thames (Figure 1). This represents a wide range of river types, in terms of water quality, flow, land use and sewage input, and covered the majority of the basin above the tidal limit. For example, mean concentrations of total phosphorus (September 2009–April 2011) range from 31 μg l^−1^ (River Leach—Le) to 700 μg l^−1^ (River Thame—Tm). Mean nitrate concentrations are generally high across the catchment owing to groundwater contamination, but range from 3.97 mg NO_3_-N l^−1^ (Enborne—En) to 19.94 mg NO_3_-N l^−1^ (the Cut—Cu). Mean flow varies considerably across the catchment, from 1.2 m^3^ s^−1^ (Enborne—En) in the tributaries to 46.8 m^3^ s^−1^ in the lower reaches of the River Thames (Runnymede—TR). Supplementary Table 1 summarises the site names and locations, and further catchment and monitoring site details are given elsewhere (Bowes *et al.*, 2012; Bowes *et al.*, 2014).

A single water sample was collected from each site by lowering a clean bucket into the centre of the river channel, transferred and subdivided into autoclaved polypropylene bottles and stored in the dark until they were taken back to the lab for filtering on the same day. Samples (0.5–1.0 l) for microbiological analysis were pre-filtered through glass fibre GF/A filters (Whatman, Buckinghamshire, UK) to reduce particulate and algal biomass, and then through a 0.22-μm Durapore membrane filter (Merck Millipore, Watford, UK) to collect microbial cells. Filters were stored at −80 °C for later analysis.

Water chemistry parameters, including pH, alkalinity, suspended sediment, soluble reactive phosphorus, total dissolved phosphorus, total phosphorus, ammonia (NH_4_), dissolved reactive silicon, fluoride (F), chloride (Cl), nitrite (NO_2_), nitrate (NO_3_), total dissolved N, sulphate (SO_4_) and dissolved organic carbon (DOC) were measured as detailed in Neal *et al.* (2012). In addition, the concentrations of a suite of metals, including sodium (Na), boron (B), iron (Fe), magnesium (Mg), zinc (Zn), copper (Cu) and aluminium (Al) were measured by inductively coupled plasma–optical emission spectrometry (ICP-OES). Site physico-chemical and biotic data is given in Supplementary Table 1.

### Flow cytometry of bacterioplankton and phytoplankton

Phytoplankton enumeration and characterisation was carried out using a dual colour flow cytometry protocol as described in Read *et al.* (2014), identifying 10 major groups of phytoplankton in the Thames, including diatoms (one group), chlorophytes (three groups), cryptophytes (two groups) and cyanobacteria (four groups). To count total bacterioplankton, samples were fixed in 2% formaldehyde for 1 h at room temperature, stored in the dark at 4 °C overnight and analysed the following day. An aliquot of 0.5 ml from each sample was stained with SYBR Green I (Sigma-Aldrich, Gillingham, UK) at a final concentration of 1:1000 for 30 min at room temperature in the dark. An addition of 2.5 μl of 1 μm diameter beads (Life Technologies, Paisley, UK) to each sample was used as a calibration and counting standard. Each sample was run for 1 min at a low flow rate (∼5 μl per min) on a Gallios flow cytometer (Beckman-Coulter, High Wycombe, UK), using excitation with a 488 nm laser. Gates were manually drawn in Kaluza 1.2 software (Beckman-Coulter) to distinguish and count both high (HNA) and low (LNA) nucleic acid bacteria.

Bacterial activity was measured by flow cytometry using the dye 5-cyano-2,3-ditolyl tetrazolium chloride (CTC) to infer bacterioplankton activity rates (Sieracki *et al.*, 1999). Briefly, a final concentration of 5 mM CTC (Sigma-Aldrich) was incubated with each water sample at room temperature for 2 h in the dark. The reaction was stopped by the addition of 0.2 μm filtered 2% (wt/vol) final concentration formaldehyde, freshly made from reagent-grade paraformaldehyde (Sigma-Aldrich). A cytogram of side scatter (SS) against FL3 (620 nm BP 30 nm) drawn in Kaluza 1.2 software (Beckman-Coulter) was used to distinguish CTC-positive cells. The percentage positive CTC cells were calculated as a proportion of the total bacterioplankton count.

### DNA extraction and sequencing

DNA was extracted from 0.22 μm membrane filters using methods described in Huang *et al.* (2009). DNA amplification and pyrosequencing were carried out at Molecular Research LP (Lubbock, TX, USA). Microbial tag-encoded FLX amplicon pyrosequencing was carried out using 16S V1-V3 spanning primers Gray28F 5′-GAGTTTGATCNTGGCTCAG-3′ and Gray519r 5′-GTNTTACNGCGGCKGCTG-3′. Initial generation of the sequencing library utilised a one-step PCR with a total of 30 cycles, a mixture of Hot Start and HotStart high fihigh fi taq polymerases, and amplicons originating and extending from the forward primers. Tag-encoded FLX amplicon pyrosequencing analyses utilised Roche 454 FLX instrument (Roche 454 Life Sciences, Branford, CT, USA) with titanium reagents.

### Data processing and analysis

Sequencing reads were demultiplexed and filtered for quality and size (reads <367 or >548 bp were discarded as possible errors) using the QIIME pipeline (Caporaso *et al.*, 2010), denoised with ACACIA (Bragg *et al.*, 2012) and chimeras were identified and removed with ChimeraSlayer (Haas *et al.*, 2011). The sequences were clustered into operational taxonomic units (OTUs) with UCLUST (Edgar, 2010) as part of the QIIME package and representative sequences were selected (pick_rep_set.py, QIIME). The taxonomy of OTUs was determined by RDP Classifier with 80% bootstrapping classification confidence (Wang *et al.*, 2007) using the Greengenes Oct 2012 database (McDonald *et al.*, 2012) and Newton freshwater 16S SSU database (Newton *et al.*, 2011). The OTU table was subsequently rarefied down to 2179 sequences per sample for comparative diversity analyses (multiple_rarefactions_even_depth.py, QIIME). A table with OTU identities from both databases is given in Supplementary Table 2. Sequences are deposited in the European Nucleotide Archive under the accession number PRJEB6879 (http://www.ebi.ac.uk/ena/data/view/PRJEB6879).

All statistical analyses were carried out in R (v.3.0.1) (R Core Team 2013) using the package ‘Vegan' v2.0–10 (Oksanen *et al.*, 2013). Environmental and flow cytometry (biotic) data were individually tested for normality (Shapiro–Wilkes test, *P*<0.05). Variables that were not normally distributed were transformed to normality or as close to normality as possible using either log or square root transformations. In order to determine the major drivers of bacterial community composition, a Bray–Curtis dissimilarity matrix generated from the OTU table was compared to three broad categories of potential drivers: spatial, environmental and biotic. These were, in turn, represented by the location of the sites within the river network, the physico-chemical variables and the flow cytometry-derived phytoplankton community. To explore the best spatial predictor of bacterial community composition, three contrasting measurements that represented site location within river catchment were compared. These were: (1) the dendritic network length (km), which is a measure of the cumulative length of the branching river network upstream of the sampling site, (2) the Euclidian distance (km) between sites, which is simply the straight line distance between sampling points and (3) the drainage catchment area (km^2^) for each site. All geographic measures were calculated using ArcGIS (Esri Ltd, Aylesbury, UK). Mantel tests and correlograms were carried out on OTU data using Bray–Curtis dissimilarity matrices to examine the strength of the relationship between each spatial predictor and bacterial composition. Function ‘Bioenv' in the R package ‘Vegan' was used to identify the subsets of environmental and phytoplankton variables that best predicted bacterial community composition (Clarke and Ainsworth, 1993). The selected subset of variables for each category was reanalysed using Mantel tests and correlograms to examine the strength of the relationship between dissimilarity matrices from each optimised group of predictors and the bacterial community composition as before.

Before calculating alpha diversity for each site, sequences were subsampled to the site with the lowest number of sequences (2179 sequences). To explore whether the freshwater component of the bacterial community behaved in a different manner to the whole community, Shannon's H index was calculated from species-level abundance tables (summarize_taxa.py, QIIME) derived from the Greengenes 16S rRNA gene database that is not habitat specific (McDonald *et al.*, 2012) or the ‘Newton database' comprising a curated selection of known freshwater microbes (Newton *et al.*, 2011). Visual examination of the relationships between the bacterial community structure and the physico-chemical and biotic parameters were assessed using non-metric multidimensional scaling using the ‘metaMDS' function in Vegan, based on dissimilarities calculated using the Bray–Curtis index. To examine the OTUs that had the highest contribution to the ordination, the function ‘Envfit' was run with 999 permutations and used to plot significantly (*P*<0.001) correlated variables. To examine relationships between the OTU ordination and the environmental and biotic variables, ‘Envfit' was again used to plot significantly (*P*<0.05) correlated variables on the ordination.

Finally, a Pearson's correlation matrix with *P*-values was generated using the R package HMISC (Harrell, 2013) and used to identify individual OTUs from the core river microbiome (present in >50% of the sites) with significant (*P*<0.05) positive or negative relationships to dendritic distance.

## Results

**Figure 2:**
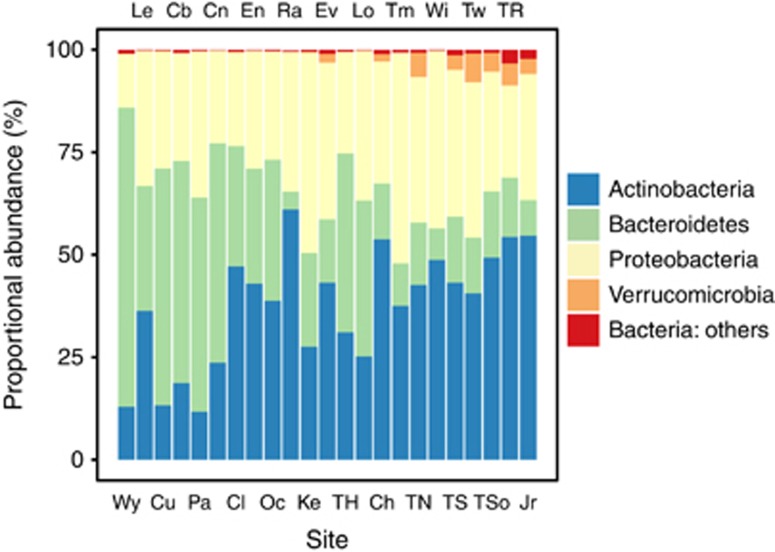
Phylum-level taxonomic composition of the bacterial community across the 23 study sites based on 16s rRNA gene sequences. The sites are placed in order of increasing dendritic distance left to right.

Sequencing generated a total of 123 068 sequences, averaging 5351 sequences per site, which were classified into 2492 distinct OTUs. Across all catchment sites, the most common phyla of bacteria were Actinobacteria, Bacteroidetes, Proteobacteria and to a lesser extent, Verrucomicrobia (Figure 2). Genus-level composition of Actinobacteria, Bacteroidetes and Alpha-, Beta- and Gammaproteobacteria are shown in bar charts in Supplementary Figures S1–S5. The most abundant OTUs were represented by species of known specialist freshwater bacteria (Greengenes classification followed by Newton database classification in parentheses), including the Actinobacteria ‘*Candidatus* Rhodoluna' (Luna1-A3), ACK-M1 (acI-A1), *Microbacteriaceae* (Luna1-A3) and ‘*Candidatus* Aquiluna rubra' (Luna1). Abundant Bacteroidetes OTUs comprised *Arcicella* (bacIII-A), *Flavobacterium* (bacII-A), *Fluviicola* (bacV) and *Chitinophagaceae* (bacI-A1). The most abundant Proteobacteria were *Limnohabitans* (Lhab-A1, Lhab-A4), *Rickettsiales* (LD12 alfV-A), *Polynucleobacter* (PnecC) and *Sphingomonadaceae* (alfIV-B). Verrucomicrobia were represented by one abundant group of OTUs; *Cerasicoccaceae* (Opitutaceae).

Our study found a clear relationship between bacterial composition at the phylum level and dendritic distance upstream (Figure 2); OTUs belonging to the phylum Actinobacteria increased in abundance within the community with increasing dendritic distance. Although making up a far smaller proportion of the community, Verrucomicrobia OTUs followed a similar pattern. Conversely, OTUs belonging to the phylum Bacteroidetes went from being the dominant phylum in sites with short dendritic distances upstream to a minor component in downstream sites (Figure 2).

**Figure 3:**
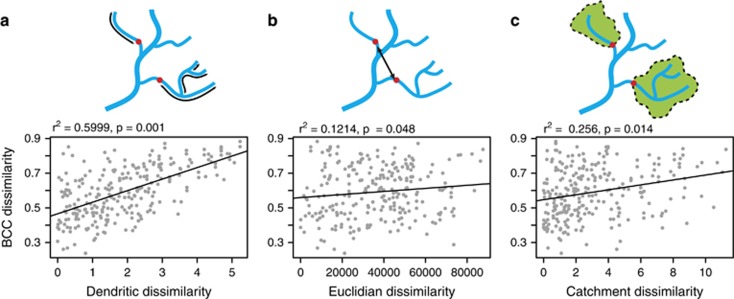
A comparison of three measures of site location against a Bray–Curtis dissimilarity matrix of bacterial community composition (BCC); cumulative dendritic distance upstream (log km) (**a**), direct site-to-site (Euclidian) distance (m) (**b**) and catchment area (log m^2^) (**c**). *r*^2^ and *P*-values refer to Mantel tests of matrix correlation.

The best spatial measurement for predicting bacterial community composition was the (log) sum of the dendritic distance upstream (Mantel *r*^2^=0.5999, *P*<0.001), followed by (log) catchment size (Mantel *r*^2^=0.256, *P*=0.014) and then Euclidian distance between sites (Mantel *r*^2^=0.1214, *P*=0.048) (Figures 3a–c). The optimal subset of environmental variables with the best correlation to the bacterial composition dissimilarity matrix contained three parameters—nitrite (NO_2_), sulphate (SO_4_) and log copper (Cu) concentrations (Bioenv correlation=0.2663) (Supplementary Figure S6A). However, this subset was not strongly correlated to the bacterial community dissimilarity matrix (Mantel *r*^2^=0.2314, *P*=0.014), especially when compared to dendritic distance upstream. The optimal biotic model contained four parameters; log group 2 Chlorophytes, group 4 pico Chlorophytes, bacterial HNA/LNA ratio and log CTC-positive bacterial cells (Bioenv correlation=0.599) (Supplementary Figure S6B). This subset of biotic variables was more strongly correlated to the bacterial community dissimilarity matrix (Mantel *r*^2^=0.5993, *P*=0.001) than the environmental subset.

**Figure 4:**
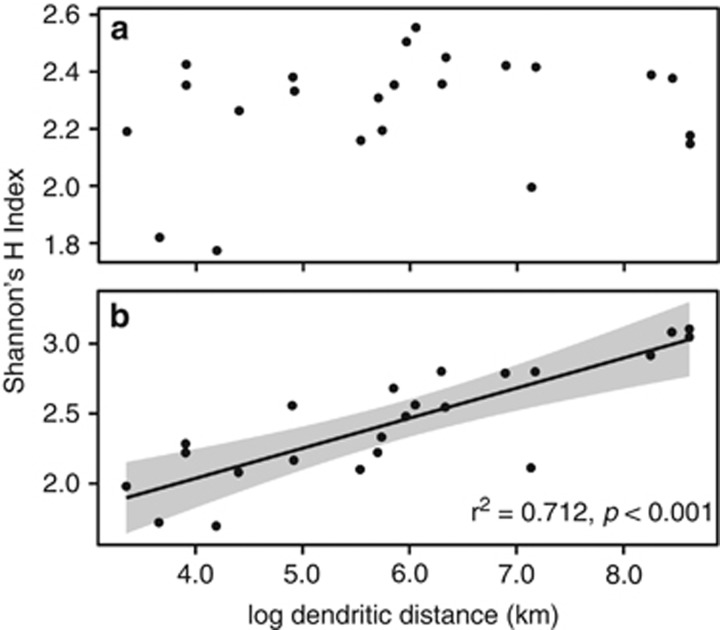
Shannon H′ diversity against log dendritic distance downstream (km) after classifying at the species-level against the Greengenes database (**a**) and the Newton database of freshwater bacteria (**b**).

There was no significant correlation between dendritic distance upstream and Shannon H′ diversity of the species-level abundance table based on the Greengenes database (Figure 4). However, there was a significant relationship between the species-level diversity (Shannon H′) of the freshwater component of the community (Newton database) and dendritic distance upstream (lm, *r*^2^=0.712, *P*<0.001). This implies that a greater number of taxa related to known freshwater taxa were found at increasing dendritic distance.

**Figure 5:**
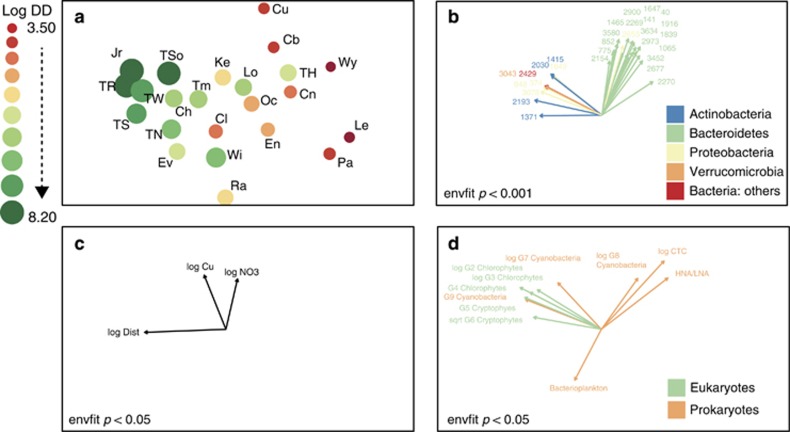
NMDS plot showing the relationship between the bacterial community compositions at each site (**a**). Point size and colour relate to the dendritic distance upstream of each site. Log DD stands for ‘log dendritic distance'. Significant OTUs (**b**), environmental, (**c**) and biotic (**d**) variables correlating with the ordination as determined by Envfit analysis.

Non-metric multidimensional scaling was used to explore the relationships between the bacterial community composition at each site, along with the biotic (phytoplankton) and environmental variables. There was separation along axis 1 between sites from headwaters (low dendritic distance) and downstream sites (high dendritic distance) (Figure 5a). Envfit analysis confirmed that downstream sites were associated with OTUs belonging to Actinobacteria, Proteobacteria and Verrucomicrobia, whereas headwater sites were strongly associated with OTUs from the phylum Bacteroidetes (Figure 5b). Only two environmental and one spatial variable were significantly (*P*<0.05) correlated with the ordination; log dendritic distance, log copper (Cu) and log nitrate (NO_3_) (Figure 5c). Three chlorophyte, two cryptophyte and one cyanobacterial group were significantly (*P*<0.05) associated with the downstream communities (Figure 5d). The percentage of CTC-positive cells and the HNA/LNA ratio were associated with the upstream sites (Figure 5d).

**Figure 6:**
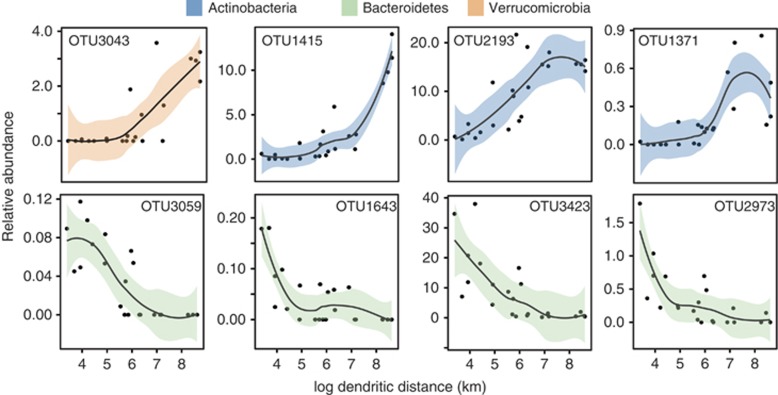
OTUs with positive and negative correlations with log dendritic distance (km) with loess curves fitted to highlight trends.

Of the nine OTUs with a significant (Pearson's *r*, *P*<0.05) negative correlation with dendritic distance, eight belonged to the phylum Bacteroidetes (the top four are shown in Figure 6). The 15 OTUs with a significant positive correlation to dendritic distance represented more taxa; 3 of the 4 most abundant OTUs belonged to Actinobacteria (Figure 6) and 1 to a Verrucomicrobia but also included Alpha and Betaproteobacteria and 2 Bacteroidetes OTUs belonging to the family Chitinophagaceae (Supplementary Table 3).

**Figure 7:**
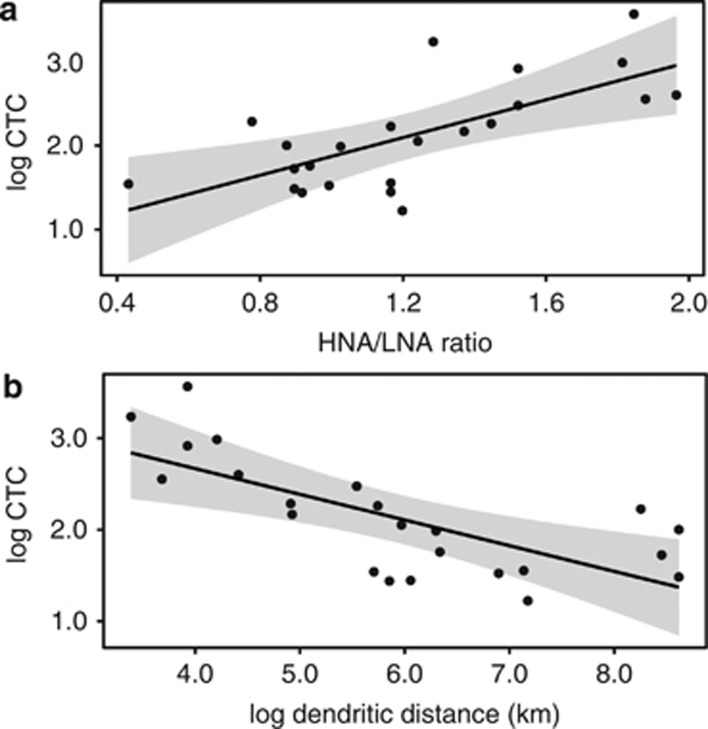
Relationship between the ratio of HNA to LNA cells and the number of log CTC-positive cells (**a**). The relationship between log dendritic distance (km) and the number of log CTC-positive cells (**b**).

Bacterial activity and viability rates, measured by reduction of the dye CTC, showed a significant positive correlation with the ratio of HNA to LNA bacteria (*r*^2^=0.4648, *P*<0.001), where a higher proportion of HNA bacteria in the community correlate with increased activity (Figure 7a). In addition, both the number of CTC-positive cells (*r*^2^=0.4933, *P*<0.001) and HNA/LNA ratio (*r*^2^=0.3437, *P*<0.05) had significant negative correlations with dendritic distance (Figure 7b).

## Discussion

Our results showed that bacterial community composition in this river basin was more related to spatial parameters than physical and chemical variables. In particular, site location (measured as cumulative river network distance upstream from the site) was found to be the most predictive spatial feature above other measures of position such as Euclidian distance and site catchment area (Figures 3a–c). This infers that ecological processes pertaining to river network length are of prime importance in driving the assemblages of bacterial communities in this river system. This contrasts with other, more static habitats such as soils and even lakes, where differences in chemical and physical parameters are known to correlate closely with differences in community assemblages between sampled sites (Lindstrom *et al.*, 2005; Griffiths *et al.*, 2011).

A succession in the composition of the bacterial community from headwaters to downstream was observed, transitioning from a Bacteroidetes- to an Actinobacteria-dominated community (Figure 2). Individual OTU abundance and their correlation with dendritic distance confirmed the observed phylum-level patterns; OTUs with the strongest positive correlations with dendritic distance were Actinobacteria and Verrucomicrobia and those OTUs with negative correlations belonged to the phylum Bacteroidetes. There is a lack of similar studies of whole river catchments with which to compare these results. However, research carried out in a subtropical river in China (Hu *et al.*, 2014), the upper reaches of the Mississippi (Staley *et al.*, 2013), a stretch of the Ohio river (Schultz *et al.*, 2013) and the mouth of the Columbia river (Fortunato *et al.*, 2013) show similarities in terms of bacterial composition; Actinobacteria, Bacteroidetes and Proteobacteria, and in some cases Cyanobacteria, Firmicutes and Verrucomicrobia make up the dominant members of riverine bacterial communities.

We propose three linked explanations for the observed pattern in bacterial community composition related to (1) water residence time, (2) changing resource availability and (3) biotic interactions in the form of top-down structuring of bacterial communities. First, we suggest that the change in community composition downstream is linked to the residence time of the water and resultant community succession. The length of the river network is correlated with water residence time, with longer networks having further and therefore more time for surface water to travel (Stewart *et al.*, 2011). As water transitions downstream, a community of bacteria better adapted to the freshwater environment (the ‘natives') has time to develop, outcompeting the transient ‘vagabond' or ‘tourist' species (Newton *et al.*, 2011; Crump *et al.*, 2012) that are washed into the watercourse. This idea is supported by the increase in abundance and diversity of known freshwater taxa (that is, from a database predominantly comprising freshwater taxa) during the headwater–downstream transition (Figure 4b).

The succession from a Bacteroidetes to Actinobacteria-dominated community may also be viewed as a succession of species of bacteria with r- and k-strategist lifestyles (Weinbauer and Hofle, 1998). The upper reaches of the river is a ‘new' environment with potentially lower levels of competition, which would favour rapidly growing species that can utilise available resources quickly (r strategists). Indeed, previously identified ‘freshwater taxa' (Newton *et al.*, 2011) in these headwater communities were notably cultivable fast-growing orders such as Flavobacteriales and Bacteroidales. Competition may become more intense downstream as the community of specialist aquatic bacteria builds in both number and complexity, resulting in k-strategist species that are more competitive and have lower growth rates and narrower niches. Freshwater Actinobacteria have previously been observed to be slower growing than other phyla (Simek *et al.*, 2006), which fits with our observation that their abundance increases downstream as residence time increases.

These findings are supported by our observation that high bacterial activity rates were observed in the tributaries and decreased in downstream sites (Figure 7b). The ratio of HNA to LNA bacteria also showed a strong longitudinal trend, with the number of LNA relative to HNA bacteria increasing downstream. As with other studies, we found that higher abundances of HNA cells were positively correlated to activity rates (Lebaron *et al.*, 2002). Examination of the correlation between HNA/LNA and OTU abundance showed that HNA was correlated with Bacteroidetes OTU abundance, and LNA was correlated to Actinobacteria OTU abundance (Supplementary Figure 7), supporting the links between community composition, activity and dendritic distance downstream. Although little information on the identities of HNA/LNA bacteria exists for freshwater systems, we note that in marine systems Bacteroidetes have been commonly identified as major components of the HNA group (Schattenhofer *et al.*, 2011; Vila-Costa *et al.*, 2012)

We were unable to identify a dominant physico-chemical driver of bacterioplankton community composition in the Thames. It is possible that the observed succession is caused by an unmeasured environmental factor; however, it is to be noted that we comprehensively measured 29 major nutrients, metals and ions in these samples. As examples, the rivers Thame (TM), Cut (Cu) and Ray (Ra) are some of the most polluted in this study in terms of major nutrient (total phosphorus and NO_3_) concentrations, but there was no evidence to suggest they shared relatively similar communities (Figure 5a). Likewise, although the ‘clean' sites on the Rivers Leach (Le) and Pang (Pa) do cluster together, the Kennet (Ke), with similar total phosphorus and NO_3_ concentrations, does not. Previous studies have identified DOC to be a significant driver of microbial community composition (Jones *et al.*, 2009; Li *et al.*, 2012) and different taxa of aquatic bacteria have varying preferences in terms of carbon utilisation (Salcher *et al.*, 2013). Yet in our study there was no strong relationship with bacterial community composition. Overall concentrations of DOC peaked at the mid dendritic distance and longer-term weekly data over the course of two years showed no clear longitudinal pattern in DOC concentration (data not shown). However, we did not measure the composition of DOC species in terms of molecular weight and complexity, and it is possible that the proportions of labile and recalcitrant pools could change downstream (Vannote *et al.*, 1980; del Giorgio and Pace, 2008; Kaplan *et al.*, 2008), thus influencing communities and in particular the proportion of r and k strategists. Although not measured in this study, particulate carbon and nitrogen have previously been found to correlate with riverine bacterial communities (Fortunato *et al.*, 2013), providing possible evidence for links between the planktonic and sediment communities.

Another candidate for the observed succession is that biotic interactions have a role in structuring the bacterial community. We found a correlation of bacterial composition with the phytoplankton community (figure 5d), suggesting other planktonic microbial communities are also changing downstream in a similar manner. The relationship between bacterioplankton and phytoplankton composition may simply reflect the same physical driver (residence time) rather than a direct ecological interaction. However, there is strong evidence that phytoplankton blooms can have a significant role in structuring the composition of bacterial communities through the input of photosynthetically derived carbon sources (Kirchman *et al.*, 1991; Teeling *et al.*, 2012), and the possibility that this is a significant driver in this system cannot be discounted. Increasing phytoplankton biomass across the River Thames basin with increasing river length and residence time (related to water transfers with adjacent canal systems) have been observed previously (Bowes *et al.*, 2012).

Another biotic interaction in the form of mortality from heterotrophic protists and/or viral lysis could have a top-down role in structuring the community. Different bacterial taxa are known to vary in their resistance to both grazing and viral lysis (Pernthaler, 2005) and predation by flagellates and ciliates have been shown to structure the composition of bacterial communities in mesocosm experiments (Salcher *et al.*, 2005). The increased residence time downstream may allow for higher levels of top-down predation to develop in the lower reaches; lags in bacterivore abundance have previously been observed in aquatic systems (Tanaka *et al.*, 1997). Microscopic observations of freshwater Actinobacteria have shown that they are generally free-living ‘ultramicrobacteria' (Hahn *et al.*, 2003), and this small size has been linked to avoidance from grazing, whereas a number of studies have indicated that members of the Bacteroidetes are vulnerable to grazing (Jurgens *et al.*, 1999; Simek *et al.*, 2001; Salcher *et al.*, 2005).

In conclusion, this study has shown that there are distinct successional changes in the composition of the bacterial community during the transition from tributaries to the lower reaches of a temperate lowland river. This change, largely driven by the changing abundance of members of the phyla Actinobacteria and Bacteroidetes, corresponds to changes in bacterial activity rates and the composition of cells in terms of their nucleic acid content. Given the lack of strong correlation with any of the river's physico-chemical variables measured, it would appear that this change is largely driven by an inherent property of lotic systems, that is, the residence time of the water. Rivers have been described as ‘lotic conveyor belts' (Schultz *et al.*, 2013) owing to the fact that, at least for planktonic organisms, movement is determined by the direction and flow of water. Because of this, the bacterial population at any one site is in a state of flux, with constant immigration from the upstream community and emigration to the downstream one. In this view, sampling sites in this study not only represent a spatial distribution but also a time series, where downstream sites with longer water residence times contain ‘older' river water and a planktonic community that is in the later stages of ecological succession. The next steps to better understand this transition are to investigate the substrate preferences, growth rates and relative contribution of other biotic interactions in structuring riverine communities. One major aspect missing from this study is information on the temporal shifts in community structure. We purposefully chose a period of basal flow for this study owing to the relative stability of the river catchment during this time. However, rivers and streams are highly dynamic systems and gaining a better understanding of the magnitude of change over time would offer key insights into the controls of bacterial communities along a river continuum. Finally, a better understanding of the functional consequences of this shift in composition would allow us to have a better understanding of the role of planktonic microbes in rivers.
