# Supplementary material for: Catchment-scale biogeography of riverine bacterioplankton
Source: ISME J. 2014 Sep 19;9(2):516–26. doi: 10.1038/ismej.2014.166 (PMC4303643; doi:10.1038/ismej.2014.166)
Supplement: Supplementary Figures S1–S7 [file ismej2014166x1.pdf]

Supplementary figure S1

Bar chart showing the relative abundance of genera within the group Actinobacteria from 454 sequence data of 16S rRNA encoding DNA. Sites are ordered with increasing upstream dentridtic distance, from left to right.

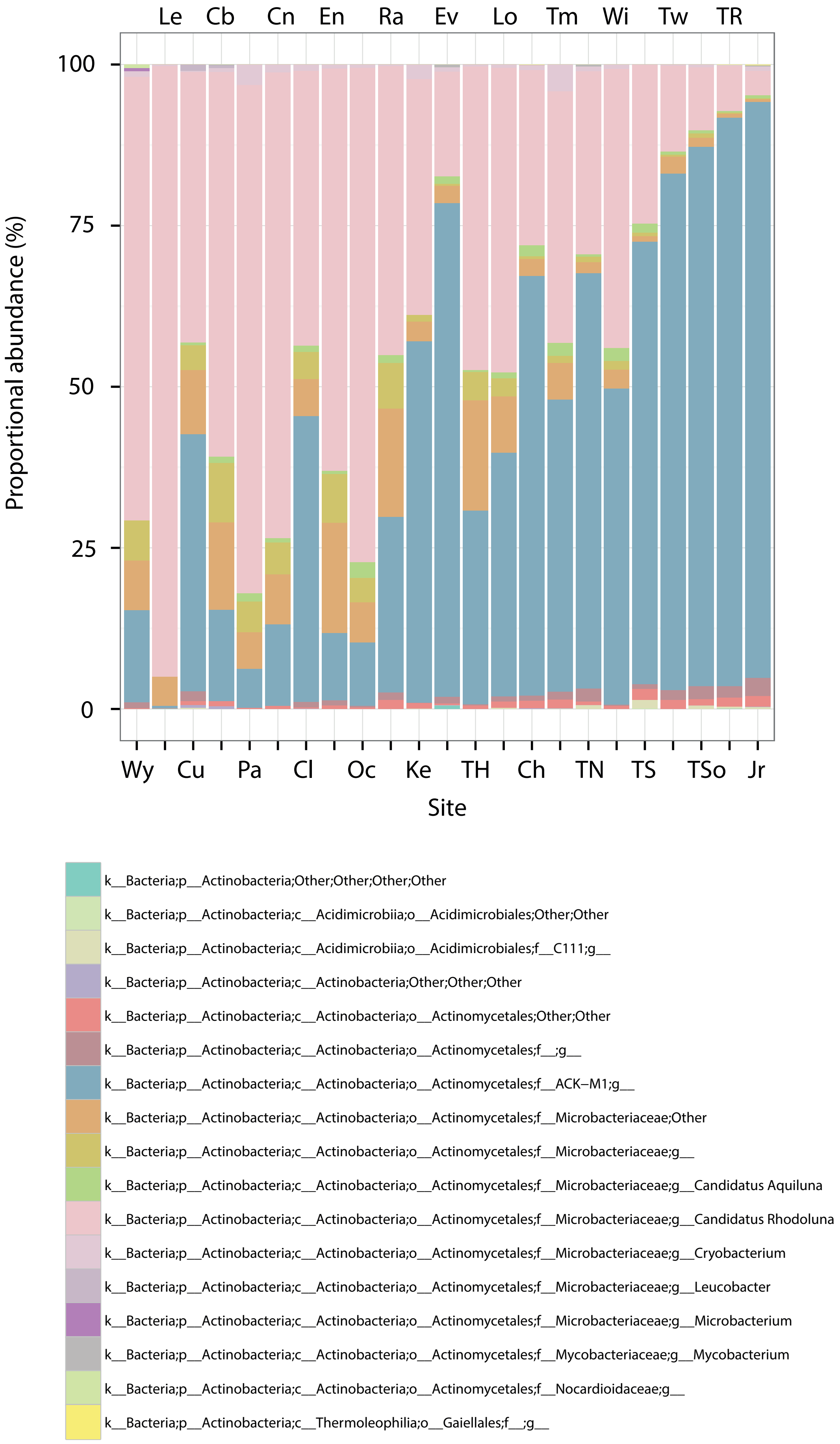

Supplementary figure S2

Bar chart showing the relative abundance of genera within the class Alphaproteobacteria from 454 sequence data of 16S rRNA encoding DNA. Sites are ordered with increasing upstream dentridtic distance, from left to right.

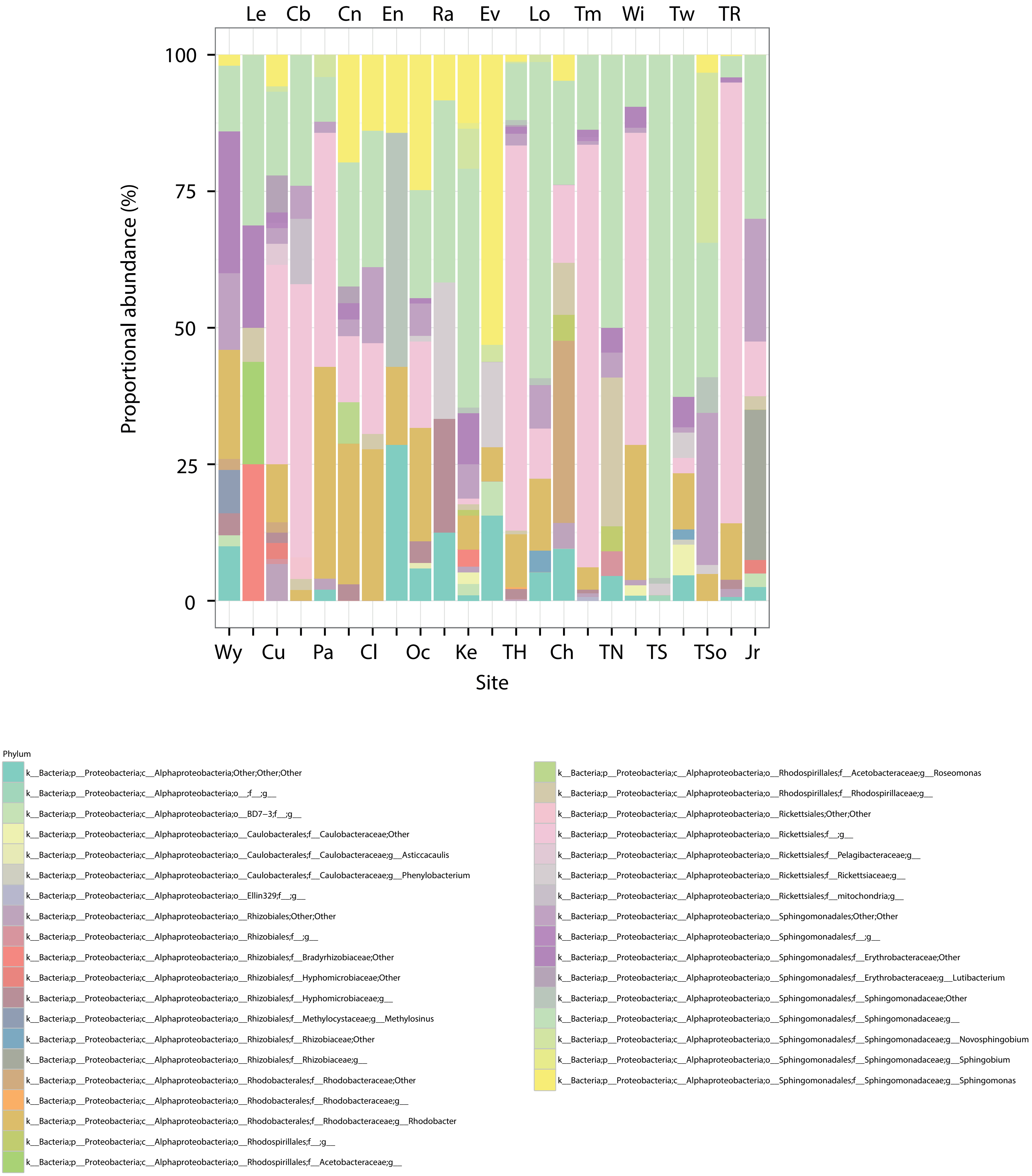

Supplementary figure S3

Bar chart showing the relative abundance of genera within the phylum Bacteroidetes from 454 sequence data of 16S rRNA encoding DNA. Sites are ordered with increasing upstream dentridtic distance, from left to right.

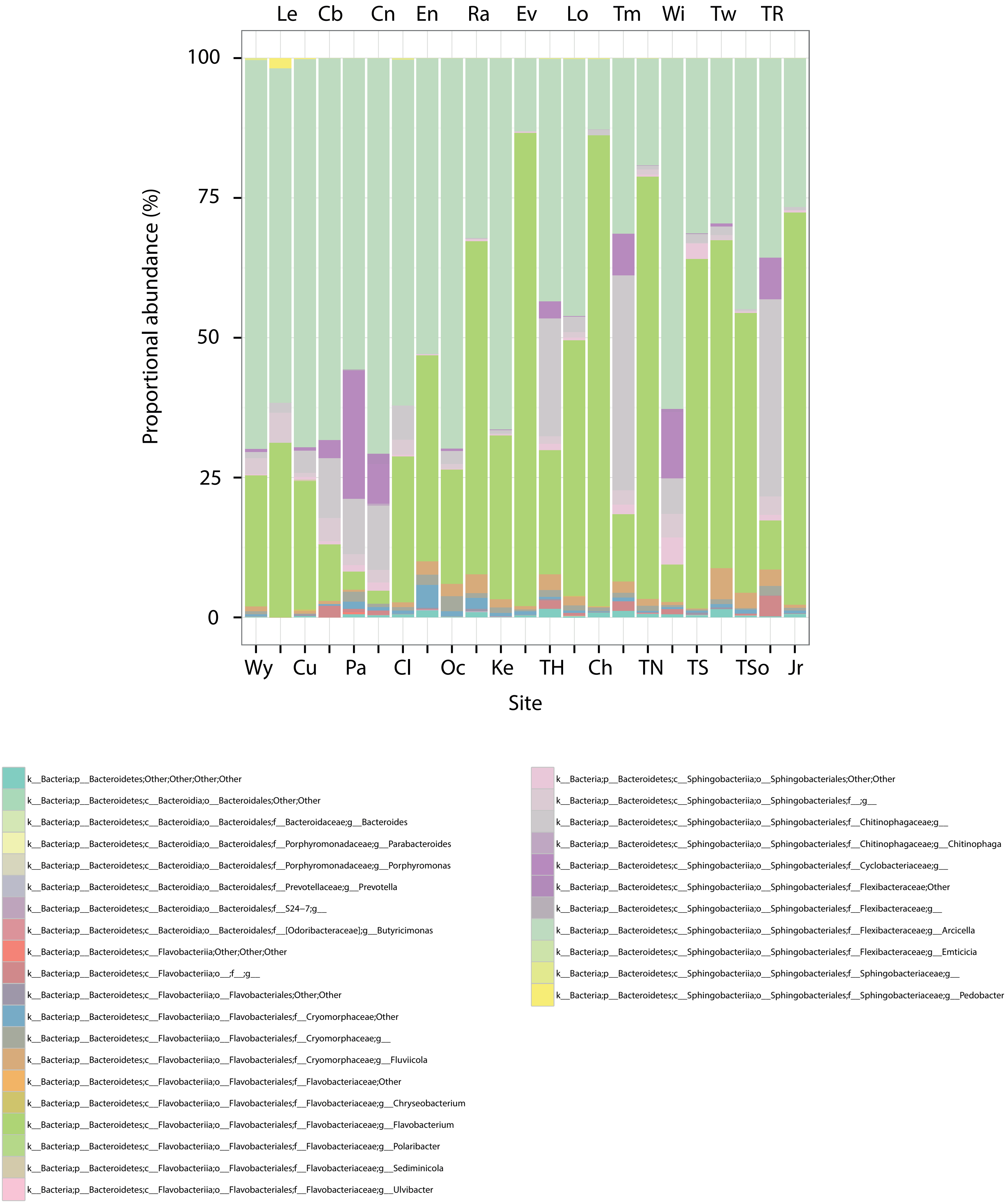

Supplementary figure S4

Bar chart showing the relative abundance of genera within the class Betaproteobacteria from 454 sequence data of 16S rRNA encoding DNA. Sites are ordered with increasing upstream dentridtic distance, from left to right.

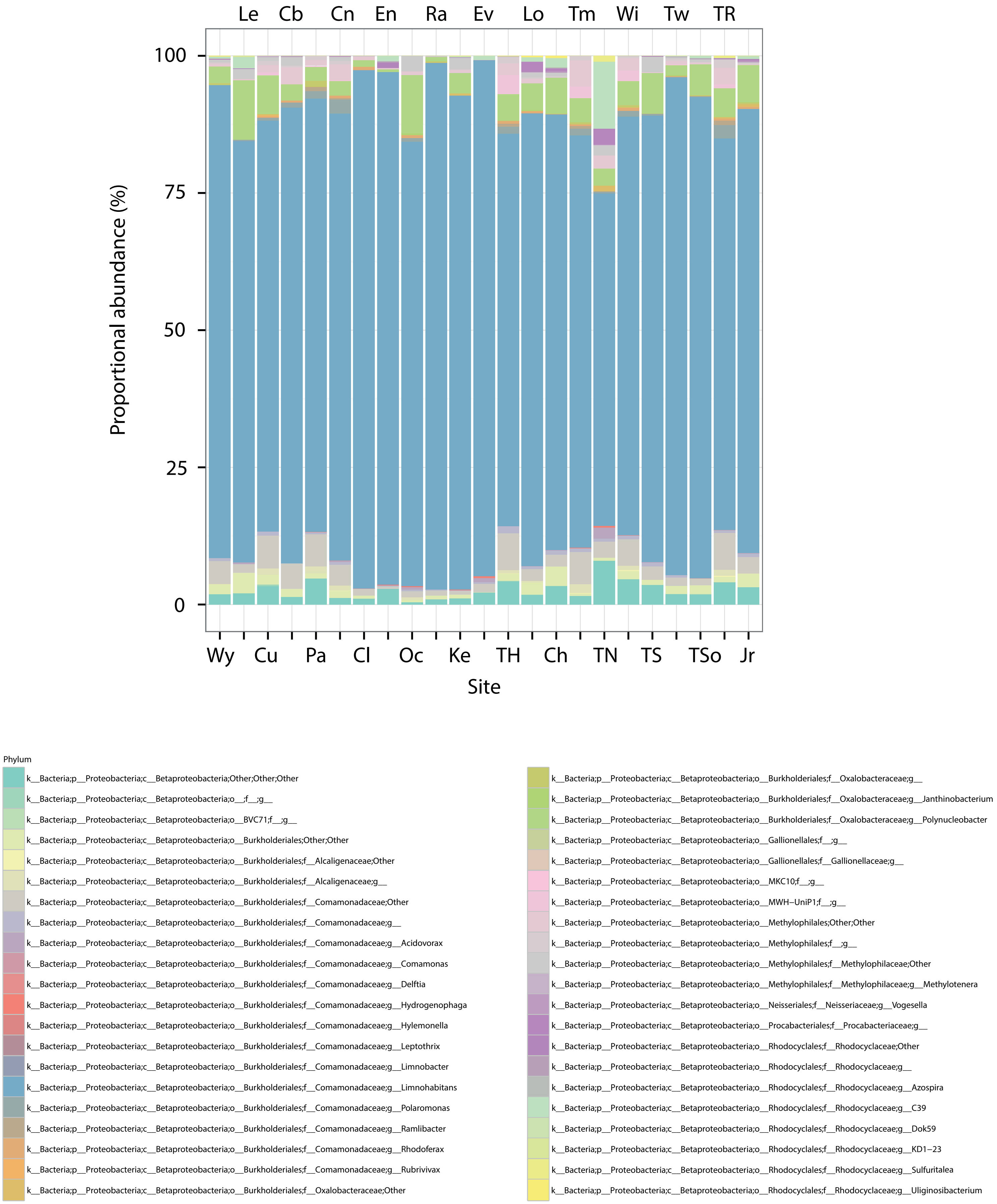

Supplementary figure S5
Bar chart showing the relative abundance of genera within the class Gammaproteobacteria from 454 sequence data of 16S rRNA encoding DNA. Sites are ordered with increasing upstream dentridtic distance, from left to right.

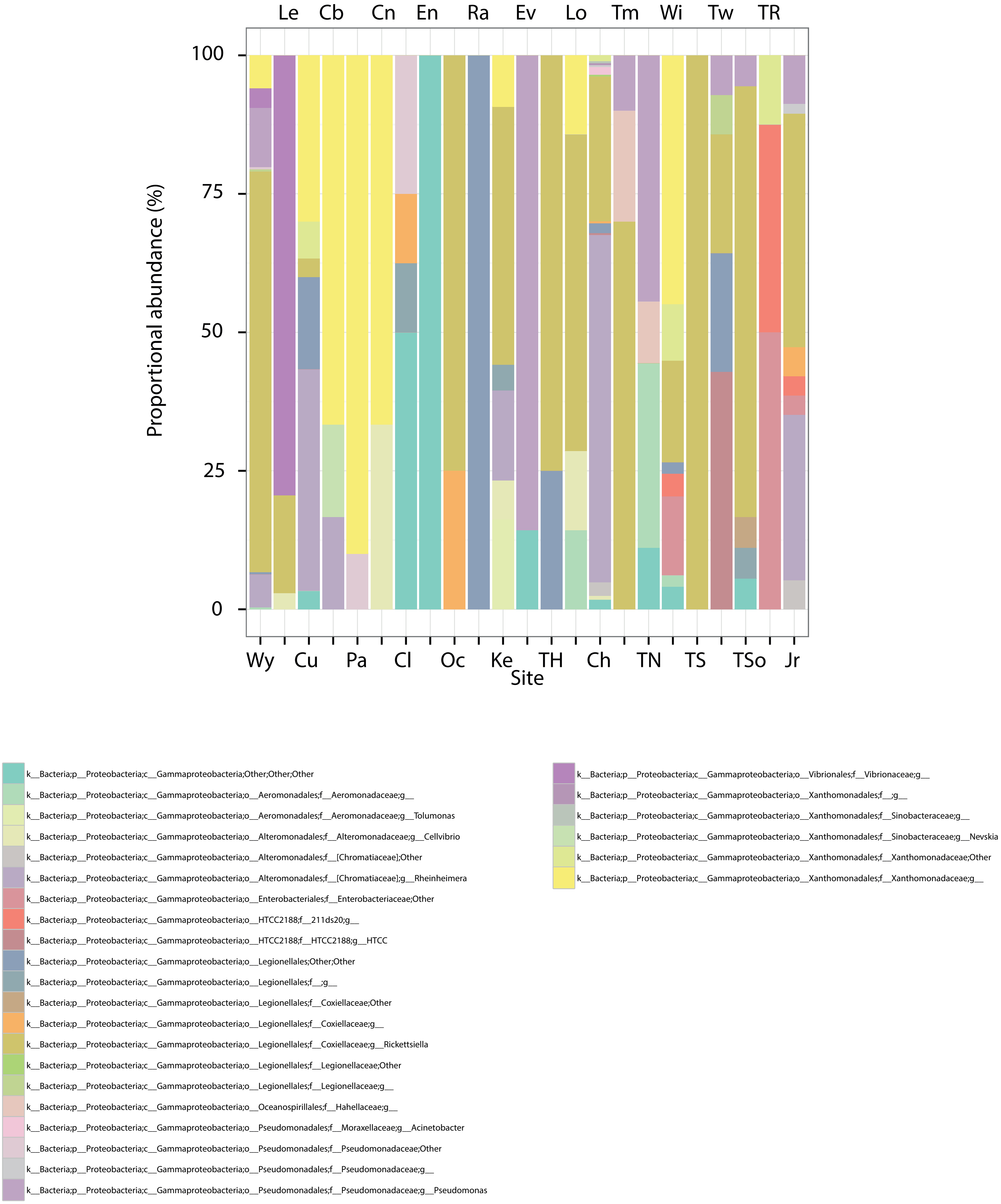

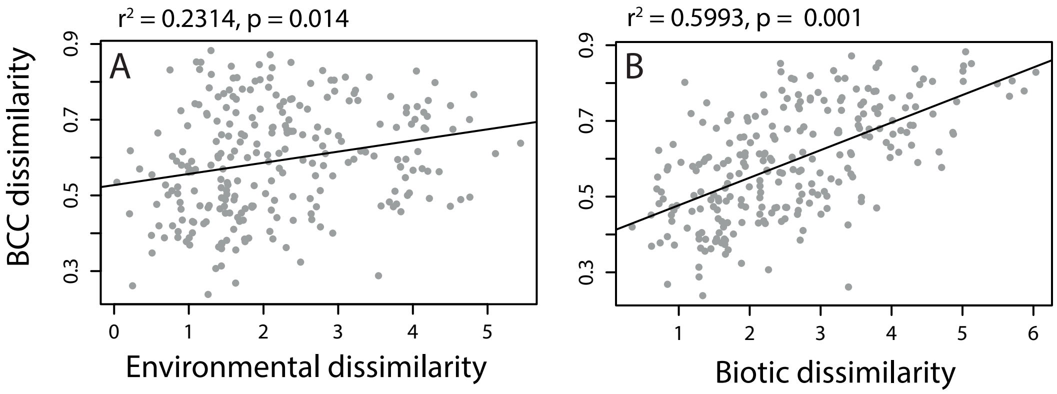

Supplementary Figure S6.
A comparison environmental (A) and biotic (B) dissimilarity matrices against a Bray-Curtis dissimilarity matrix of bacterial composition. R^0 and p values refer to Mantel tests of matrix correlation.

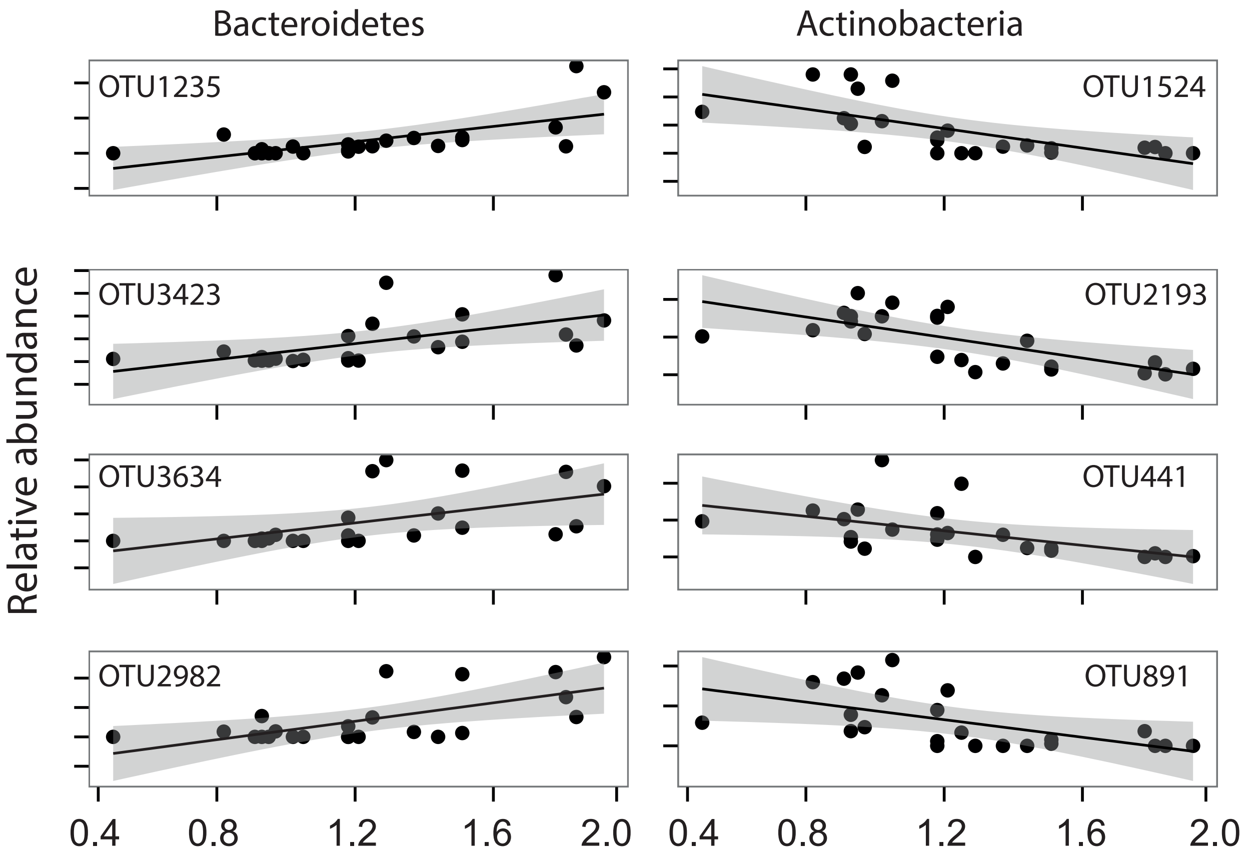

Supplementary Figure S7.
Scatter plots showing the Operational Taxonomic Units (OTUs) with the highest pearson's r correlation to the ratio of High Nucleic Acid (HNA) and Low Nucleic Acid (LNA) bacteria.
