# Supplementary material for: Catchment-scale biogeography of riverine bacterioplankton
Source: ISME J. 2014 Sep 19;9(2):516–26. doi: 10.1038/ismej.2014.166 (PMC4303643; doi:10.1038/ismej.2014.166)
Supplement: Supplementary Table 3 [file ismej2014166x4.doc]

**Supplementary Table 3.**

OTUs with significant positive and negative correlations with log dendritic distance upstream as determined by Pearson correlation coefficient (please see main text for method details).

| OTU | Phylum | Class | Order | Family | Genus | Species | R-value | P-value |
| --- | --- | --- | --- | --- | --- | --- | --- | --- |
| 12 | p__Actinobacteria | c__Actinobacteria | o__Actinomycetales | f__ACK-M1 | g__ | s__ | 0.890341 | 1.27E-08 |
| 257 | p__Verrucomicrobia | c__Opitutae | o__[Cerasicoccales] | f__[Cerasicoccaceae] | g__ | s__ | 0.763845 | 2.22E-05 |
| 10 | p__Actinobacteria | c__Actinobacteria | o__Actinomycetales | Other | Other | Other | 0.749527 | 3.84E-05 |
| 11 | p__Actinobacteria | c__Actinobacteria | o__Actinomycetales | f__ | g__ | s__ | 0.749188 | 3.89E-05 |
| 131 | p__Proteobacteria | c__Alphaproteobacteria | o__Rhodobacterales | f__Rhodobacteraceae | g__Rhodobacter | s__ | 0.736158 | 6.22E-05 |
| 49 | p__Bacteroidetes | c__Sphingobacteriia | o__Sphingobacteriales | f__Chitinophagaceae | g__ | s__ | 0.717202 | 0.000117 |
| 137 | p__Proteobacteria | c__Alphaproteobacteria | o__Rickettsiales | f__ | g__ | s__ | 0.715828 | 0.000123 |
| 188 | p__Proteobacteria | c__Betaproteobacteria | o__Methylophilales | f__ | g__ | s__ | 0.712308 | 0.000137 |
| 159 | p__Proteobacteria | c__Betaproteobacteria | o__Burkholderiales | f__Comamonadaceae | Other | Other | 0.693143 | 0.000246 |
| 158 | p__Proteobacteria | c__Betaproteobacteria | o__Burkholderiales | f__Alcaligenaceae | g__ | s__ | 0.680914 | 0.000348 |
| 187 | p__Proteobacteria | c__Betaproteobacteria | o__Methylophilales | Other | Other | Other | 0.67476 | 0.000413 |
| 173 | p__Proteobacteria | c__Betaproteobacteria | o__Burkholderiales | f__Comamonadaceae | g__Polaromonas | s__ | 0.620225 | 0.001593 |
| 51 | p__Bacteroidetes | c__Sphingobacteriia | o__Sphingobacteriales | f__Cyclobacteriaceae | g__ | s__ | 0.508285 | 0.013271 |
| 175 | p__Proteobacteria | c__Betaproteobacteria | o__Burkholderiales | f__Comamonadaceae | g__Polaromonas | s__ | 0.491472 | 0.017233 |
| 2 | Other | Other | Other | Other | Other | Other | 0.473967 | 0.022327 |
| 38 | p__Bacteroidetes | c__Flavobacteriia | o__Flavobacteriales | f__Cryomorphaceae | g__Fluviicola | s__ | -0.41504 | 0.048917 |
| 16 | p__Actinobacteria | c__Actinobacteria | o__Actinomycetales | f__Microbacteriaceae | g__Candidatus Rhodoluna | s__ | -0.4249 | 0.043276 |
| 172 | p__Proteobacteria | c__Betaproteobacteria | o__Burkholderiales | f__Comamonadaceae | g__Limnohabitans | s__curvus | -0.42974 | 0.040702 |
| 54 | p__Bacteroidetes | c__Sphingobacteriia | o__Sphingobacteriales | f__Flexibacteraceae | g__Arcicella | s__ | -0.507 | 0.013544 |
| 36 | p__Bacteroidetes | c__Flavobacteriia | o__Flavobacteriales | f__Cryomorphaceae | Other | Other | -0.52007 | 0.010965 |
| 37 | p__Bacteroidetes | c__Flavobacteriia | o__Flavobacteriales | f__Cryomorphaceae | g__ | s__ | -0.53768 | 0.008141 |
| 25 | p__Bacteroidetes | Other | Other | Other | Other | Other | -0.56156 | 0.005299 |
| 42 | p__Bacteroidetes | c__Flavobacteriia | o__Flavobacteriales | f__Flavobacteriaceae | g__Flavobacterium | s__ | -0.5946 | 0.002769 |
| 41 | p__Bacteroidetes | c__Flavobacteriia | o__Flavobacteriales | f__Flavobacteriaceae | g__Flavobacterium | Other | -0.71587 | 0.000123 |
